# COVID-19′s Impact on American Women’s Food Insecurity Foreshadows Vulnerabilities to Climate Change

**DOI:** 10.3390/ijerph18136867

**Published:** 2021-06-26

**Authors:** Maryruth Belsey-Priebe, Deborah Lyons, Jonathan J. Buonocore

**Affiliations:** 1 Department of International Relations, Harvard University Extension School, Emeryville, CA 94608, USA; 2 Department of Sustainability, Salt Lake City Corporation, Salt Lake City, UT 84111, USA; debbie.lyons@slcgov.com; 3 Center for Climate, Health, and the Global Environment, Harvard T.H. Chan School of Public Health, Boston, MA 02215, USA; jbuonocore@mail.harvard.edu

**Keywords:** gender, climate change, food security, COVID-19

## Abstract

The COVID-19 pandemic is wreaking havoc on human lives and the global economy, laying bare existing inequities, and galvanizing large numbers to call for change. Women are feeling the effects of this crisis more than others. This paper explores the pre-COVID relationships and amplified negative feedback loops between American women’s economic insecurity, lack of safety, and food insecurity. We then examine how COVID-19 is interacting with these intersecting risks and demonstrate how climate change will likely similarly intensify these feedback loops. The COVID-19 pandemic may be revealing vulnerabilities that societies will face in the wake of an increasingly warming world. It is also an opportunity to build resilience, inclusiveness, and equity into our future, and can help inform how to include gender equity in both COVID-19 and climate recovery policies. Finally, we identify possible strategies to build resilience, specifically highlighting that gendered economic empowerment may create a buffer against environmental health hazards and discuss how these strategies could be integrated into a women-centered Green New Deal.

## 1. Introduction

The COVID-19 pandemic is wreaking havoc on the global economy and human lives around the world, laying bare existing inequities and galvanizing large numbers to call for change. Since some are feeling the effects of this crisis more than others, women chief among them, it is important to understand causal factors in order to mitigate the disproportionate impacts of future crises on vulnerable populations. The following analysis will survey several issues, including climate change, gender inequity and gender-based violence, food insecurity, economic insecurity, the COVID-19 pandemic, and the intersection between these issues. It will explore how these factors interact and amplify each other, and then compare the impacts of the COVID-19 pandemic to the impacts of accelerated climate warming on these health and economic factors. The synthesis of the existing literature will demonstrate many similar risks and vulnerabilities between climate change and the pandemic and show how these stressors can amplify negative feedback loops. Finally, this paper will present several potential policy solutions that may disrupt these harmful feedback loops, such as women’s economic empowerment, and possibly informing a women-focused Green New Deal. The COVID-19 pandemic could be a dark harbinger of what societies will face in the wake of an increasingly warm world. It is also an opportunity to examine the inequitable impacts of major societal disruption, and then use these lessons to build resilience, inclusiveness, and equity into a future with climate change through programs such as a women-centered Green New Deal.

## 2. A Vicious Cycle: Food Insecurity and Gender Inequity in the U.S.

### U.S. National Trends in Sexism and Women’s Food Insecurity 

**Figure 1:**
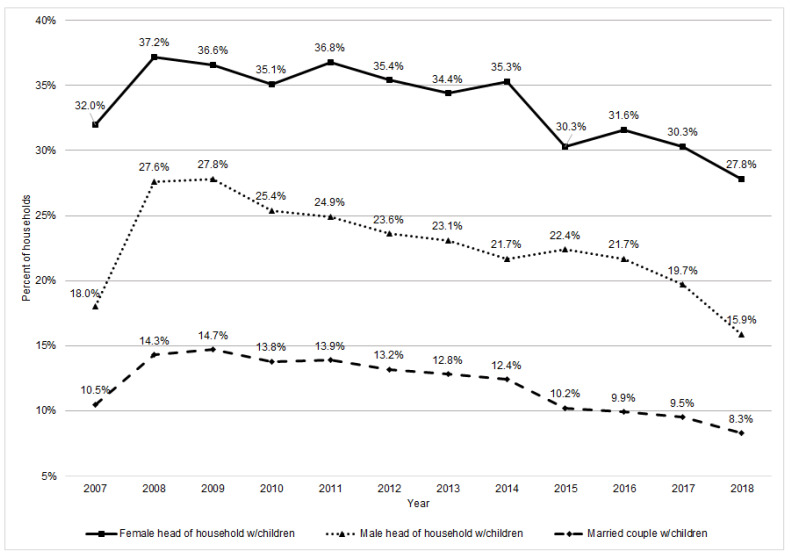
Percent of households with children facing food insecurity, by household type (female head, male head, married) and by year from 2007 to 2018 (Adapted from ref. [3]).

Food insecurity, measured by the distance someone has to travel to obtain food, and how much food they can afford, is felt more acutely by women [1]. A systematic review assessing household food insecurity (primarily in the Americas and Europe) found that women were 40 percent more likely to report food insecurity, and that female-headed households were 75 percent more likely to be food insecure than male-headed households [2]. U.S. households with children have higher food insecurity (13.9 percent) than those without (7.5 percent), and since women are more likely to head single parent households, they are at higher risk [3]. American female-headed households with children are the second most food insecure (Figure 1), with food insecurity rates at 27.8 percent compared to only 15.9 percent for male-headed households with children. U.S. households with incomes below 185 percent of the poverty threshold are most food insecure at 29.1 percent [3]. 

Despite a general improvement in gender equity, many sexist norms that impact economic and food insecurity still persist in America [4]. Traditional gender roles often limit the opportunity for women to hold paying jobs outside of the home, making financial independence for some impossible. According to a national-level measure of gender equity, the U.S. ranks 51st out of 149 countries, with lower participation rates for women in economic leadership and higher rates of part-time jobs and unpaid daily work hours [5], factors that may play a role in gendered food insecurity. Additionally, in many households globally, women and girls may be given smaller food portions because of the way food is divided along gender lines [4]. What these statistics show is that American women continue to face disproportionate levels of food insecurity due largely to prevailing sexist gender norms.

## 3. The Economic Factors That Predispose Women to Food Insecurity

The impacts that crises such as the COVID-19 pandemic and climate change have on world food security are felt differently depending on where you live and who you are. Since women already experience greater food insecurity, they are likely more vulnerable to disruption [2]. American women are economically disadvantaged because of gender discrimination [6]. If pandemics increase in frequency and as climate change worsens, existing gender wage gaps will likely deepen, women’s poverty rates will likely intensify, and factors such as increased gender-based violence and family responsibilities will strain women’s earning potential and abilities to afford and prepare healthy foods.

### Broader National Gendered Wage and Benefits Gaps 

Despite gains over the past several decades, today, American women are paid less than their male counterparts for the same jobs. Women without higher education earn only 52 percent as much as men without higher education [6]. While women with higher levels of education manage to close the gap more, they still earn only 73 percent that of similarly educated men [6]. Other studies find that if mothers change employers after giving birth, they pay an additional −7.1 percent wage penalty by being segregated into lower-paying jobs [7].

A more hidden disadvantage is that women receive fewer benefits, such as pension plans, paid leave, and health insurance. Researchers have found that although the gender gaps for wages and benefits have narrowed over time (the benefits gap narrowing more quickly), overall benefits rates have fallen for both genders [8]. Even accounting for reduced benefits, women earn only 76 percent as much as men, and receive 82 percent of the health insurance benefits, 76 percent of paid leave benefits, and 67 percent of pension benefits [8]. Since women take on the majority of unpaid care work for elderly adults and children, they often find themselves choosing between taking unpaid time off or neglecting the needs of family members [9]. The differences are even more stark for women of color [8]; the average time spent daily on unpaid household and care work is 7.2 h for Hispanic women (compared to 3.7 for Hispanic men), 6 h for Asian women (3.5 for Asian men), 5.5 h for White women (3.8 for White men), and 5.1 h for Black women (2.7 for Black men) [10]. Ultimately, these choices hamper women’s employment success and are leading reasons women quit their jobs, retire early, or involuntarily reduce their hours, all of which add additional economic strains [9]. 

To compensate for lower income and benefit levels, women often take on multiple jobs. According to the U.S. Census Bureau, 8.8 percent of women hold at least two jobs, while 8 percent of men do [11]. Women are also more likely than men to hold only part-time jobs, most of which do not come with benefits [11]. The U.S. Department of Labor recognizes that greater transparency in overall compensation would level the playing field for working women, yet most employers do not disclose gender-disaggregated pay and benefit levels [12]. These economic factors, including women’s uncompensated and often hidden care work contributions, deepen the food insecurity women face because of sexist cultural norms. 

These economic limitations make it difficult for low-income women to afford a healthy mix of foods for themselves and their children due to lower incomes and increased cost of higher quality food. One Canadian study of 2731 fifth grade students calculated the actual cost difference between foods of various qualities; the researchers looked at the correlation between diet quality, measured by the Diet Quality Index-International (DQI), and food costs [13]. They found that for every one-unit increase in DQI, the diet cost per student increased by CAD $0.07 per day [13]. Diets that met recommendations for fruits, vegetables, meats, and protein alternatives (combined) were CAD $1.92 higher per day than those that did not meet these recommendations [13]. This indicates that the financial burden of choosing healthier foods can be significant, especially for families with multiple children. As such, many women opt for lower quantities of food, lower quality foods, or both. As food becomes scarce in a household, women also tend to reduce their personal food consumption relative to other family members, putting their personal health at risk [14]. This evidence demonstrates that American women’s income is lower, their employment benefits are smaller, which puts further constraints on the household budget, and their unpaid family care responsibilities are higher, which limits their income-earning abilities. Consequently, American women, especially women of color, experience higher levels of food insecurity than their male counterparts.

## 4. The Role Crises Play in Women’s Food Insecurity

Global crises such as poverty, pandemics, and climate change will likely stress economies, food systems, and family relationships in ways that will increase the food insecurity of women.

### 4.1. Economic Shocks and Food Insecurity

#### 4.1.1. Broader Trends Linking Economic Shocks and Food Insecurity 

Existing economic and food insecurity gender gaps generally widen during global crises such as the COVID-19 pandemic [15]. History suggests that the types of economic shocks from climate change will disproportionately impact women’s earning potential [15]. One study of historical wage trends in the U.S. found that male–female wage gaps are counter-cyclical [15] in that they are negatively correlated to the overall the economy. When the unemployment rate is low, there is greater competition for workers; this lowers discrimination by employers, allowing more women to gain jobs, especially higher paying ones [15]. However, when unemployment is higher, such as during major market shocks or economic depressions, there is an increase in pure wage discrimination against women, where women receive lower wages [15]. During periods of high unemployment, there are more available workers, allowing employers to favor men over women, resulting in increased levels of discrimination against women [15]. For instance, the overall unemployment rate in the U.S. was 6.01 percent between 1979 and 2009, but the wage disadvantage *for women* during that same period was 11 percent, even after accounting for state and year differentials [15]. When the 2008 financial collapse hit, women experienced higher rates of unemployment [15]. During the period following the financial collapse, the average unemployment rate increased by 4.7 percentage-points, but the wage disadvantage for women increased by 5 percent, with the wage gap increasing from 11 percent to 16 percent [15]. The impact was greater for women overall than for Hispanics or for Black Americans during this period [15]. American women’s lower income levels increase their vulnerability to greater food insecurity during times of crisis.

#### 4.1.2. The COVID-19 Pandemic’s Economic Shock and the Impact on American Women’s Food Insecurity 

On a global level, women make up 39 percent of people employed, but accounted for 54 percent of job losses as of May 2020 [16]. By February 2021, the COVID-19 pandemic resulted in millions of jobs lost in the U.S. [17]. According to the Institute for Women’s Policy Research, while American men and women were on approximately equal footing in terms of unemployment rates before the pandemic, since then, women have lost 5.4 million jobs, while men lost only 4.4 million [18]. In December 2020, the National Women’s Law Center found that 100 percent of the jobs lost in the U.S. were women’s jobs, and an estimated 2.1 million women have left the labor market entirely since the start of the pandemic [19]. The jobs women lost were primarily in the government, retail, and hospitality sectors—most of which are women-dominated [19]. Researchers also point out that higher demands for women to fill unpaid caregiving roles have compounded these job loss trends [20]. According to the Bureau of Labor Statistics January Economics News Release, the reality for women of color is even more stark. While White men and White women had unemployment rates of 5.5 and 5.1 percent, respectively, Black women’s unemployment rate was 8.5 percent [21] and the unemployment rate among Hispanic women was 8.8 percent [22]. The racial disparities are likely due to the fact that many Black, Hispanic, and Native American workers are employed in restaurants, retail, and hospitality. Globally, progress has been made over the past few decades on gender equity, but economic experts warn that progress could be reversed if policymakers do not act quickly to protect women [23]. 

During the COVID-19 pandemic, food insecurity in the U.S. has been on the rise along with unemployment rates, with women, veterans, and Black, Hispanic, and Native Americans seeing the greatest increases in food insecurity [24,25]. Record numbers of people are seeking food assistance at food banks, and Feeding America estimates that the largest food bank network in the country will see a USD 1.4 billion shortfall [26]. The rate of households with children under 18 living characterized by food insecurity increased by 130 percent from 2018 to May 2020 [27]. Given how intimately children’s food security is tied to maternal income, this likely points to increases in women’s food insecurity as well [27]. In Los Angeles County, women were found to be 57 percent of those reporting food insecurity [28], with even higher rates reported by women of color. This evidence, therefore, demonstrates that, as with other crises, the COVID-19 pandemic has had a disproportionate impact on the income and food security of American women.

#### 4.1.3. COVID-19 and Climate Parallels: Global Economic Shocks and Food Insecurity 

As with the COVID-19 pandemic, climate change is expected to cause economic shocks and increase food insecurity. Rising sea levels, declining crop and fisheries outputs, extreme weather events such as floods and hurricanes, and the health effects (physical and mental) of increased temperatures on the productivity of the labor pool will all have deleterious impacts on the global economy [29]. One estimate found that a 3 °C temperature increase could result in global warming costs of USD 9.6 trillion to GDP annually, or approximately 3 percent of 2100 world GDP [30]. In another analysis, the financial costs of failing to transition the economy off of fossil fuels at USD 20 trillion, which is approximately the size of the U.S. annual economic output [31]. For comparison, as of June 2020, the pandemic had already cost the global economy USD 9 trillion [32] and may rise to as high as USD 82 trillion [33]. Despite these projections, a Rainforest Action Network analysis of bank financing for fossil fuels found that the 37 biggest world banks have invested USD 2.7 trillion since 2016 [34]. Knowing that economic wellbeing is closely tied to food security, we can expect that these market shocks will exacerbate American women’s existing food insecurities.

#### 4.1.4. Expected Climate Impacts on the Economy in the U.S.

A 2020 analysis of 1500 regions in 77 states around the world has projected that unless significant steps are taken to reduce global warming emissions, the U.S. economy could shrink between 10 and 20 percent by 2100, with other regions of the world shrinking more than 20 percent [35]. A first-of-its-kind report on managing climate risk in the U.S. economy by the Commodity Futures Trading Commission (CFTC) noted that, “Climate change poses a major risk to the stability of the U.S. financial system and to its ability to sustain the American economy” [36]. It goes on to explain that the risks to the U.S. financial system are complex, systemic, and in many cases, unknown, with the potential for multiple factors to interact simultaneously and in short timeframes to ricochet shocks and stresses from the financial system into the real economy, creating spillover disruptions that impact low-income and people of color in particular [36]. In the food system, the report notes that, “Sub-systemic shocks related to climate change can undermine the financial health of community banks, agricultural banks, or local insurance markets, leaving small businesses, farmers, and households without access to critical financial services” [36].

Beyond the financial system, natural disasters are expected to put significant strain on the U.S. economy. In an Environmental Defense Fund analysis, all extreme weather event types since 1980 have increased fourfold (hurricanes have increased sevenfold), costing Americans USD 1.75 trillion [37]. The report also notes that USD 1 billion-level natural disasters have impacted all 50 states, and without action to reverse climate change, every 1 °C of warming is expected to cost the U.S. economy 1.2 percent of GDP, the equivalent of USD 257 billion annually (in 2019 terms) [37]. Combined, economists expect that financial stressors and natural disasters will further limit household income and wealth for the most vulnerable communities [38]. 

Forced migration will also be a reality within the U.S. Hurricane Katrina was one of the most recent instances of such movements, after which there was a 60 percent decrease in women-headed households in New Orleans, especially Black American women and women with children under the age of 18. Researchers found these women did not return home to New Orleans because of lack of employment opportunities, and the high costs of housing and healthcare, showing effectively that poor women fell into a poverty trap that permanently displaced them from their homes [39]. Clearly, the risks of climate change to the U.S. economy, on both national and household levels, are significant. As the world warms, Americans are likely to experience disruptions in financial markets—disruptions that are likely to impact food systems and American food security levels, particularly for economically disadvantaged women.

### 4.2. Fragile Food Systems and Food Insecurity 

#### 4.2.1. Broader Trends Linking Food System Fragility and Food Insecurity during the COVID-19 Pandemic

Over the past 70 years, the expansion of food systems through increased “industrial farming methods, specialization, and international trade” has created inequities that have become obvious in the face of lockdowns to prevent the spread of COVID-19, illness among food production workers, and significant job losses [40]. The cracks in the system are due in part to global interconnectedness of food production and consumption. According to the Food and Agriculture Organization of the UN (FAO), 20 percent of dietary energy supply came from imported food between 1995 and 2015 [41]. The briefing outlines a whole web of systems that have been stressed. Restaurants, cafes, and cafeterias have closed or have limited service (responsible for 30 percent of all calories consumed), pushing people to eat from home or go hungry [41]. Packaging changes from industrial-size to household-size have created production and distribution disruptions as well as transport bottlenecks [41]. Demand declines have led fishers to throw back catches and farmers to let crops rot, resulting in America’s food-to-waste ratio to increase from 30 percent to 40 percent in 2020 [41]. Available credit has declined due to slow operations, which has limited cash that could be lent elsewhere [41]. National-level panic has led to export limitations and calories traded [41]. Combined, these stressors and many more have decreased the availability of food and put food system livelihoods at risk in a global recession. 

Price volatility has also plagued world food markets. Unlike previous economic crises that saw cereal prices drop sharply and remain low, the pandemic created sharp cereal price declines in the first few months, and then increased in August 2020 to 2 percent higher than the year prior [40]. However, unlike previous crises, this one created sharp price declines in sugar, dairy, meat, and vegetable oil [40]. More importantly, there has been wide variation in price fluctuations between nations, with those that rely heavily on food imports (most of which are developing) seeing the highest food price increases [42]. Given that a majority of the world’s agricultural workforce is women [43] who face a gender pay gap as high as 40 percent [44], these price shocks will be particularly damaging to their livelihoods.

Despite global coordination to stave off the worst of the disaster through the World Trade Organization and other international financial institutions, the World Food Programme (WFP) has estimated that the pandemic will add 130 million people to the existing 135 million food insecure people worldwide [45]. Looking forward, researchers expect continued pursuit of standard industrial modes of food production and trade on world markets will likely not improve agricultural livelihoods enough to resolve uneven food market dynamics [40]. These disruptions to the global food system would be expected to impact American women more acutely.

#### 4.2.2. National Changes in Food Systems during the COVID-19 Pandemic

Similar patterns are being seen in the American farms that grow primarily corn and soybeans, most of which is used to feed beef cattle, a two-tracked food system that feeds into mostly restaurants and supermarkets. The pandemic not only closed restaurants—it caused virus outbreaks in meat processing plants, causing disruptions in both crop and cattle farm production [46]. Farmers left food to rot in fields, dumped milk [47], and euthanized their animals [48]. Farm workers, particularly hired and migrant agricultural laborers, have also faced a significantly higher death rate during the pandemic [46]. Though scientists are unsure of the reason for higher mortality rates among farm workers, they conclude that these kinds of global crises create disruptions in food systems due to lost labor and agricultural output [46]. Meanwhile, thousands of the 3 million American farmers found themselves fearing hunger and homelessness [49], 30 percent of whom are women [50]. In particular, the economic impacts of the pandemic have disproportionately impacted Native American farmers, 50 percent of whom are women [51]. As this evidence suggests, farmworker health has an impact on the productivity levels of global food systems, even those in developed countries such as the U.S. where the price of food has a greater impact on women’s food security. 

#### 4.2.3. The COVID-19 Pandemic and Climate Parallels: Global Food System Fragility

As with a pandemic, climate change is expected to cause disruptions in food systems. However, beyond the food distribution problems seen during the COVID-19 pandemic, climate change will cause additional systematic complications. In particular, electricity losses will add to food spoilage and shortages, and rising temperatures will disrupt agricultural labor and subsequently lower product availability [52].

Climate change is projected to decrease global food production and intensify food insecurity due to rising temperatures, precipitation changes, and possibly through other mechanisms, including pests [4]. One model estimates that in the next 20 years, there is a 10 percent chance that global maize yields will decrease 10 percent and a 5 percent chance that wheat yields will decrease 10 percent [53]. Increases in surface water temperatures; extreme weather event frequency, intensity, and seasonality; ocean acidification; ocean reef impairment; and sea level rise are expected to negatively impact fisheries and aquaculture [54]. As one of the primary omega-3 fatty acid sources for 3.2 billion people globally, including many Americans, drops in fish catches will likely have a negative impact on human health [54].

The nutritional quality of food is also expected to deteriorate. Protein, iron, and zinc content in many food crops will likely decline by 3 percent to 17 percent if CO2 concentrations reach 550 ppm [55]. Globally, this could leave an additional 175 million people zinc deficient, 122 million protein deficient, and 1.4 billion children and childbearing-age women iron deficient (57 percent of these groups), largely in Africa, Asia, and Central and South America [55]. This reduction in nutrient density will require humans to consume more food to meet basic nutritional requirements, raising the financial burden of adequate nutrition worldwide.

Combined, disruptions linked to climate change are expected to trigger rising food prices and fluctuations, similar to those seen in the COVID-19 pandemic. Most climate models project cereal price increases between 1 percent and 29 percent (median 7 percent), even when controlling for beneficial technological changes, land-use policy adjustments, and other factors [4]. The price increases will be especially sharp for rice and coarse grains, while the price projections for wheat are likely to increase as well [4] (p. 462). Decreased food production, nutrient value losses, and volatile food prices will create an unstable food system, compounding existing food insecurities.

#### 4.2.4. Expected Climate Impacts on Food Systems in U.S.

The U.S. food system will be impacted in many of the same ways that global food systems will be impacted. Short-term disruptions in supply chains will be seen because of increased natural disasters throughout the country, including wildfires, hurricanes, and record-breaking floods. In many rural and remote island and Native American communities, disruptions to food supplies is expected to be particularly acute [52].

Additionally, there will be significant changes to U.S. agriculture systems. The Fourth National Climate Assessment report produced by 13 U.S. federal agencies for the U.S. Global Change Research Program (USGCRP) explains that rising temperatures in the Southern Great Plains will lead to increased aridity due to drying soils and increased evapotranspiration [52]. Food production facilities that rely heavily on energy and water may face cost increases and resource depletion that decrease economic viability [52]. Extreme heat will impair both worker capacity and food production, and make habitats more suitable to vectors that carry diseases such as dengue, Zika, and chikungunya [52]. In the Southeast, extreme heat will also increase livestock stress and require either new or enhanced adaptive strategies or migration to cooler climates [52]. Climate scientists have already recorded output reductions in maize, soybeans, and wheat on American farms [56].

Climate-related changes to oceans and coastal areas will also impact the U.S. food system. Ocean acidification, rising water temperatures, retreating arctic sea ice, and high storm surges resulting from a warmer planet will put ocean and marine species at risk and reduce the productivity of many fisheries [52]. Climate-caused extreme drought and changes to riparian habitats have already resulted in downturns in the wildlife- and fish-related industries, with particular losses in waterfowl, crab, and oysters [52]. In 2016, an algal bloom in the Northwest resulted in declines in the shellfish industry, increasing food bank demand by 25 percent [52]. As with disruptions in the global food system, the changes to American agricultural outputs will have an unequal impact on American women who are already food insecure.

### 4.3. Gender-Based Violence and Food Insecurity

#### 4.3.1. Broader Gender-Based Violence and Food Insecurity Linkages

In addition to the economic stresses of gender discrimination, women often face an increase in gender-based violence (GBV) during crises. On its own, violence against women can increase women’s food insecurity. In one study looking at the impacts of mortgage foreclosures during the 2008 Great Recession, researchers found that, “for every additional foreclosure per 1000 owner-occupied homes with a mortgage, family violence incidences increased by an average of 2 percent”, suggesting that foreclosure rates are a positive predictor in rates of family violence [57]. 

GBV seriously impacts emotional and physical wellbeing, hampers ability to work, reduces women’s total productive years as workers and caregivers, and consequently, deepens women’s economic challenges [58]. Women who experience intimate-partner violence are much more likely to miss work due to poor health, have difficulty remembering and concentrating on tasks, and have difficulty doing errands on their own [59]. Domestic violence may also lead to women eating less or lower quality foods in order to avoid reprisal-type violence [60,61]. Given that the pandemic has required much of the world’s population to shelter at home, millions of women and girls have been locked in homes with abusive family members, causing a sharp increase in GBV that has been referred to as the shadow pandemic within the pandemic [62]. According to a meta-analysis of studies performed since the start of the pandemic, calls from women victims of intimate partner violence increased in countries around the world, including Italy, France, India, Peru, Brazil, Canada, China, and the U.S., with calls increasing anywhere from 30 percent to 74.5 percent [63]. These factors feed into an impossible cycle: economic insecurity increases rates of GBV and food insecurity, GBV increases economic and food insecurity, and food insecurity increases rates of GBV. 

#### 4.3.2. U.S. GBV and Food Insecurity Trends during the COVID-19 Pandemic

The CDC’s National Intimate Partner Violence Survey 2010–2012 State Report shows that 37.3 percent of U.S. women over the age of 18 reported experiencing rape, stalking, or physical violence [64]. Various U.S. states have reported increases in domestic violence since the pandemic began, ranging from a 21 to 35 percent increase [64]. Studies have shown that pandemic stressors have tended to exacerbate rates of GBV among communities of color in particular [65]. Importantly, fewer than 40 percent of women who experience sexual-based violence report such crimes, suggesting that numbers are much higher [66]. Knowing that GBV tends to increase levels of food insecurity, it is likely one of the factors that have increased American women’s food insecurity during the pandemic.

#### 4.3.3. The COVID-19 Pandemic and Climate Parallels: GBV

Historically, domestic violence calls increase following natural disasters within U.S. borders as well. Intimate partner violence has been reported after disasters ranging from Hurricane Katrina [67], with rates of tripling in the year following [67], to the eruption of Mt. Saint Helens, which increased domestic violence calls by 46 percent [68], to the Loma Prieta earthquake [69], after which sexual assault rates rose 300 percent and temporary restraining orders rose 50 percent [70]. As experts have noted, no country will be immune to disasters stemming from climate change, and likewise, there is no reason to believe the U.S. will be immune to climate-related increases in gender-based violence and the food insecurity it intensifies [71].

## 5. How a Women-Centered Green New Deal Could Protect against the Worst Food Security Shocks of Climate Change 

**Figure 2:**
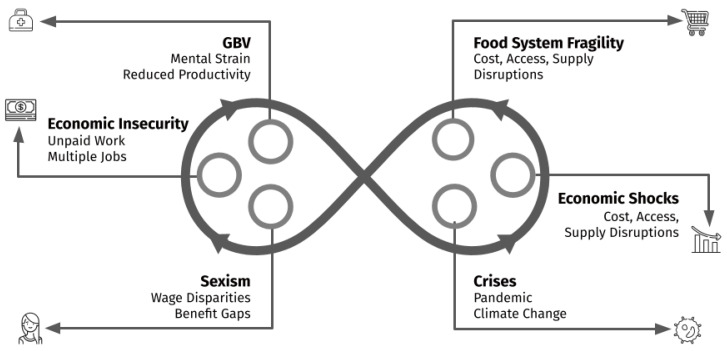
Illustration of a vicious cycle: Crises such as the COVID-19 pandemic and climate change amplify negative feedback loops between sexism, American women’s economic insecurity, and GBV, leading to deepened food insecurity.

Variations of a Green New Deal have surfaced in political discussions for some time. In 2019, U.S. Representatives Alexandria Ocasio-Cortez (New York) and Ed Markey (Massachusetts) proposed a nonbinding resolution calling for a Green New Deal [72]. The resolution aims to address climate change, create jobs, and boost the economy [73]. Given the increased vulnerabilities that exist for American women stemming from climate change, sexist norms, and economic disparities that lead to food insecurity (see Figure 2), a women-centered Green New Deal could provide support for women’s economic resilience. There is evidence that American women’s entrepreneurship can insulate them against the impacts that climate change will have on their food insecurity, through economic spillover, increased economic resilience, and decreased vulnerability to GBV. 

### 5.1. The Spillover Effect: When Women Entrepreneurs Shrink the Pay Gap

As noted, women, especially single mothers, earn less than men, which is arguably a core issue for American women’s food insecurity. Women often start their own enterprises as a way to close the pay and benefits gaps; some women business owners remunerate themselves at higher rates than if they were employed by someone else [74]. By paying themselves more, women entrepreneurs may eliminate the need to hold down multiple jobs, possibly giving them more income to purchase higher quality food, and more time to prepare meals with higher nutrient density, benefitting their children and other family members under their care. In this way, the success of women entrepreneurs has a multiplier effect: it sets in motion a chain of positive impacts that benefit the women themselves, and also their families and communities [75]. Children of mother-entrepreneurs have also been shown to cope with life challenges better and make more impactful contributions to society and in the workforce [75], thereby compounding the spillover benefits. 

### 5.2. Women Entrepreneurs Can Weather Economic Shocks Better

However, women entrepreneurs must overcome many barriers in order to achieve these benefits. For instance, survival of a new enterprise through an economic crisis depends on several factors, such as: the nature of the business, the length of time it has operated, and opportunity recognition (the ability to proactively conceptualize new business opportunities). Enterprises that demonstrate greater levels of innovation are likely to fare best during these types of setbacks, making the prospects of women-owned businesses dependent largely on individual circumstances [76]. Since women tend to use higher levels of innovation than their male counterparts to develop novel products of interest to consumers, this bodes well for their resilience during times of crisis, and ability to alleviate the societal impact of a crisis [77]. Without intervention, existing economic and cultural structures may continue to disadvantage women during future economic downturns. For example, following the 2008 Great Recession, women-led firms had more difficulty than men-led firms in securing financing in 2009 and 2010 [78]. 

### 5.3. Economic Empowerment’s Impact on Gender-Based Violence

Finally, since any societal shock—economic, health, or climatic—will likely increase rates of GBV, a Green New Deal would be strengthened with gender equity in mind to ensure that women are more resilient against these shocks. Studies have shown that when women are economically empowered using programs based on best practices, they are less vulnerable to all types of domestic violence [79]. Between 1997 and 2010, 9 percent of the decline in domestic violence in the U.S. can be explained by declining gender wage gaps, suggesting that policies that increase gender equity in resource distribution and decrease male–female gender gaps also reduce domestic violence against women [80]. 

### 5.4. Policy Recommendations

COVID-19 and many other major societal disruptions often have disproportionate impacts on women, as described above. Often, these impacts come about by exacerbating pre-existing inequities, which deepens food insecurity. Many possible interventions can be taken by governments at local, state, and federal levels, or by private institutions to mitigate the inequitable impact of disruption, especially those focused on women’s economic empowerment, such as the following:

- During the COVID-19 pandemic, women lost their jobs at higher rates than men, exacerbated by sexist norms, part-time employment, and market-sensitive industries in which women often work. Climate-related market disruptions are also expected to impact women’s economic wellbeing. Boosting economic security can help prevent women’s food insecurity, especially when she controls her wages and benefits as a small business owner. To mitigate women’s financial vulnerabilities, policies and new funding sources should be established to support women’s entrepreneurship, along with coaching on the importance of implementing gender and racial parity within their own businesses.
- Support systems such as child and elder care services were disrupted during the COVID-19 pandemic. Since women are primarily responsible for caring for dependent family members, this led to millions of American women leaving their jobs, resulting in greater economic hardship and food insecurity. We expect to see similar impacts of future climate crises without policies that recognize the value placed on the invisible, unpaid, and undervalued work that women do, such as housework, raising children, and elder care [81]. One policy solution would be to create a pension credit system for anyone, woman or man, who does any kind of unpaid caregiving work [82]. Another solution is to boost the non-wage benefits all workers receive, but especially those that would cover traditionally unpaid care-work such as paid sick leave, longer parental leave, paid time off for caregiving, and employer-sponsored health insurance, regardless of full- or part-time employment.
- Transparency has been recognized as a mechanism for increasing gender pay and benefits equity. Therefore, national policies that expand and bolster existing systems for tracking and reporting gender-disaggregated pay and benefits data should be developed. This will raise awareness of compensation, making it possible for employees to advocate for adherence to gender pay equity laws and to broaden the definition of “equal” work to include “similar” work.

## 6. Conclusions: Policies Aimed to Bolster Economic Security for Women Will Increase Women’s Resilience and Improve Food Security

Sexist norms, economic inequalities, and gender-based violence increase women’s food insecurity. The COVID-19 pandemic is highlighting ways that U.S. women, especially those with existing food insecurity, may be exceptionally vulnerable to further food insecurity due to other crises such as climate change. A women-centered Green New Deal, which includes support for women entrepreneurs, compensation for unpaid care work, and greater pay and benefits transparency, could improve women’s economic resilience and lower the vulnerability to greater levels of food insecurities as the planet warms. While the COVID-19 pandemic has highlighted many systemic vulnerabilities that will likely worsen due to climate change, the COVID-19 pandemic recovery offers opportunities for remaking the U.S. economy into one that is more just, equal, and resilient.
